# Combined Mutation And Rearrangement Screening by Quantitative PCR High-Resolution Melting: Is It Relevant for Hereditary Recurrent Fever Genes?

**DOI:** 10.1371/journal.pone.0014096

**Published:** 2010-11-23

**Authors:** Nathalie Pallares-Ruiz, Laurent Philibert, Bruno Dumont, Aurélie Fabre, Laurence Cuisset, Elodie Cointin, Cécile Rittore, Stéphan Soler, Isabelle Touitou

**Affiliations:** 1 Unité Médicale des Maladies Auto-Inflammatoires, Laboratoire de Génétique, Hôpital A de Villeneuve, Montpellier, France; 2 Assistance Publique Hôpitaux de Paris, Hôpital Cochin et Institut Cochin, Université Paris Descartes, INSERM, Paris, France; 3 Université Montpellier 1, Montpellier, France; Duke University Medical Center, United States of America

## Abstract

The recent identification of genes implicated in hereditary recurrent fevers has allowed their specific diagnosis. So far however, only punctual mutations have been identified and a significant number of patients remain with no genetic confirmation of their disease after routine molecular approaches such as sequencing. The possible involvement of sequence rearrangements in these patients has only been examined in familial Mediterranean fever and was found to be unlikely. To assess the existence of larger genetic alterations in 3 other concerned genes, *MVK* (Mevalonate kinase), *NLRP3* (Nod like receptor family, pyrin domain containing 3) and *TNFRSF1A* (TNF receptor superfamily 1A), we adapted the qPCR-HRM method to study possible intragenic deletions and duplications. This single-tube approach, combining both qualitative (mutations) and quantitative (rearrangement) screening, has proven effective in Lynch syndrome diagnosis. Using this approach, we studied 113 unselected (prospective group) and 88 selected (retrospective group) patients and identified no intragenic rearrangements in the 3 genes. Only qualitative alterations were found with a sensitivity similar to that obtained using classical molecular techniques for screening punctual mutations. Our results support that deleterious copy number alterations in *MVK*, *NLRP3* and *TNFRSF1A* are rare or absent from the mutational spectrum of hereditary recurrent fevers, and demonstrate that a routine combined method such as qPCR-HRM provides no further help in genetic diagnosis. However, quantitative approaches such as qPCR or SQF-PCR did prove to be quick and effective and could still be useful after non contributory punctual mutation screening in the presence of clinically evocative signs.

## Introduction

The hereditary recurrent fever syndromes are a group of auto-inflammatory disorders characterized by self-limited episodes of fever accompanied by inflammation of the serosal membranes, without apparent infectious etiology [1]. Included in this group are familial Mediterranean fever (FMF) and mevalonate kinase deficiencies (MKD; MIM#610377 for mevalonic aciduria and 260920 for hyper-IgD syndrome), all of which are recessively inherited. Dominantly inherited syndromes have also been described, such as TNF receptor associated periodic syndrome (TRAPS; MIM# 142680) and cryopyrin associated periodic syndromes (CAPS; MIM#120100 for familial cold autoinflammatory syndrome 1, 191900 for Muckle-Wells syndrome and 607115 for CINCA syndrome) [2].

The vast majority (FMF 97%; CAPS 96%; TRAPS 95%; MKD 81%) of the mutations implicated in the hereditary recurrent fever syndromes are punctual substitutions, and have been recorded in the dedicated online database INFEVERS (http://fmf.igh.cnrs.fr/ISSAID/infevers/). However, most laboratories use molecular techniques such as sequence analysis or exon screening (DGGE, Denaturing Gradient Gel Electrophoresis, dHLPC, denaturing high performance chromatography, HRM, High Resolution Melting analysis) that only allow for the identification of micro-rearrangements. On the other hand, a significant number of apparently mutation-free patients show evocative symptoms of hereditary recurrent fevers. The possible involvement of sequence rearrangements in these diseases has only been examined in FMF and was found to be unlikely [3]. Several methodological approaches have been have been described to search for such gene alterations: SQF-PCR, semi-quantitative fluorescent PCR [4]; MLPA®, multiplex ligation-dependent probe amplification [5]; MAPH, multiplex amplification probe hybridization [5]; qPCR, quantitative PCR [6]. More recently, innovative approaches have been developed that allow the simultaneous, rapid and accurate detection of both micro and macro-rearrangements in genes responsible for hereditary cancers. This strategy, called qPCR-HRM, is based on a real time PCR assay (gene dosage quantitative analysis) combined with HRM curves (qualitative analysis of punctual mutations) in a single tube [7].

To search for sequence rearrangements in our patients, we adapted the qPCR-HRM method for the study of intragenic deletions and duplications in the *MVK* (Mevalonate kinase, MIM#251170; NM_000431.1), *NLRP3* (Nod like receptor family, pyrin domain containing 3, MIM#606416; NM_004895.3) and *TNFRSF1A* (TNF receptor superfamily 1A, MIM#191190; NM_001065.2) genes, responsible for MKD, CAPS and TRAPS respectively. We used SQF-PCR as a confirmatory approach.

## Materials and Methods

### Study group

The study includes a total of 201 patients referred for the genetic diagnosis of hereditary recurrent fevers in our reference laboratory specialized in auto-inflammatory disorders (Montpellier, France). Informed consent was obtained from all participating subjects.

**Figure 1:**
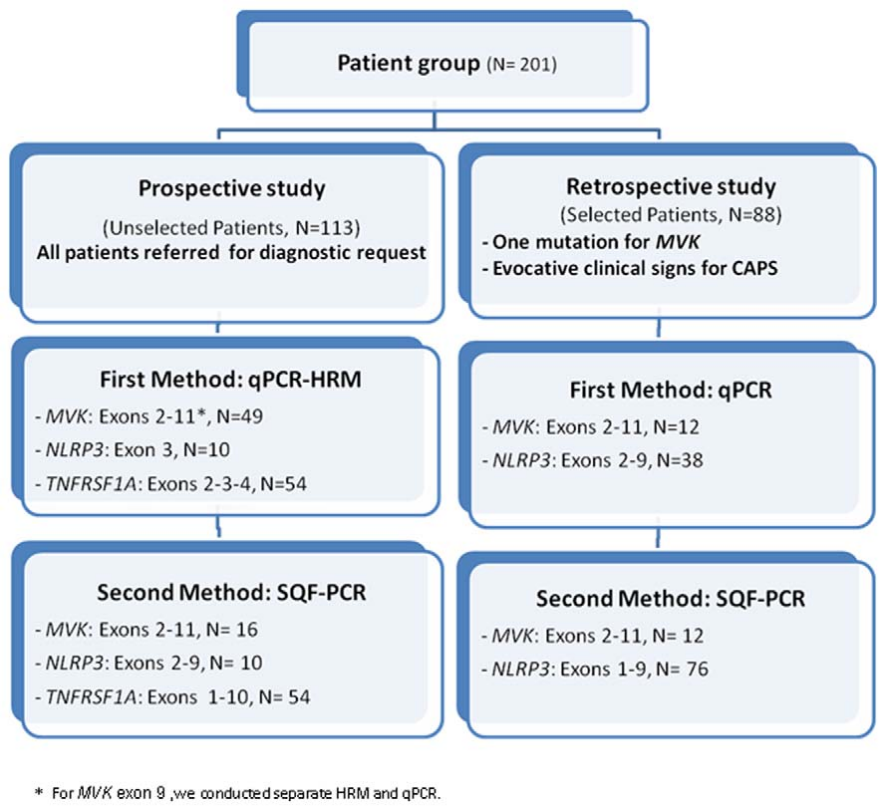
Flow chart for the screening of mutations in the genes *MVK*, *NLRP3* and *TNFRSF1A*. In our patient cohort, we performed a simultaneous search for punctual mutations and gene rearrangements using qPCR-HRM in the prospective group and qPCR alone in the retrospective group (as these patients had already been assessed for qualitative alterations), then SQF-PCR in both groups as a confirmatory quantitative approach.

The study design consisted in the delineation of two groups of patients (summarized in Figure 1). The prospective study included patients unselectively recruited over a period of 6 months in 2009 (N = 113). The retrospective study included 88 selected patients previously genotyped using our routine diagnosis strategy (screening by sequencing and/or DGGE of hot spot mutations, i.e. *MVK*, all exons; *NLRP3*: exon 3). The selection criteria for MKD (N = 12) was the detection of only a single mutation and for CAPS (N = 76), it was evocative signs, i.e. recurrent fever, urticaria, arthralgia and sometimes neurosensorial deficiency. No TRAPS patients were retrospectively selected since the clinical spectrum for this disease is too unspecific.

### DNA extraction

Human genomic DNA was prepared from whole-blood samples using the QIAmp DNA blood kit (Qiagen, Courtaboeuf, France) according to the manufacturer's standard protocol. The quality and concentration of the DNA was assessed using a Nanodrop® spectrophotometer (Coleman Technologies, Orlando, FL). DNA working solutions were prepared at a concentration of 5ng/ul for qPCR-HRM and qPCR, and 50ng/ul for SQF-PCR.

### qPCR-HRM and qPCR

#### Primer design

Common primers were used for qPCR-HRM and qPCR for the screening of exons containing mutation hot spots. Specific primers were designed for the other exons (retrospective study). Since effective primer design is an important component of HRM analysis, all amplicons were chosen to contain a single melting domain as assessed using the Poland program (http://www.biophys.uni-duesseldorf.de/local/POLAND/poland.html [8]. The PrimerBlast software (NCBI, http://www.ncbi.nlm.nih.gov/tools/primer-blast/, date of access 03/20/2010) was used to design primers with the same melting temperature (60°C), and to result in amplicons ranging between 100 and 400 bp in size. All primer sequences used in this work are listed in Table 1. The primer pairs were validated if they matched the following conditions: PCR efficiency range between 80–100% as determined using a DNA concentration gradient (40ng-20ng-10ng-5ng-2.5ng), Cycle threshold (Ct) lower than 30 cycles, and all pathological genotypes detected in the validation set.

**Table 1:** Primers used in this study.

	Gene/Exon	Forward Primer	Reverse Primer	Amplicon size (bp)	Multiplex set
qPCR-HRM^1^					
	B2M/2	cactgaaaaagatgagtatgcc	Aacattccctgacaatccc	231	
	DMD/16	tctatgcaaatgagcaaatacacgc	ggtatcactaacctgtgctgtactc	290	
	MVK/2	tcttcctgctggttctgaca	gcatcagggagaaggggta	203	
	MVK/3	tcaccctcaggcttattgct	tggtttctcctccttgcact	210	
	MVK/4	ccctctcacccacttgtgtt	aggctgaatctggactccttc	226	
	MVK/5	cgggagagtcacgtttcac	gacactggccaggtaaggac	221	
	MVK/6	ccactcctcactgccacag	ctcttgggcacctaccattg	221	
	MVK/7	cctctctcccaagtagcacag	ccctgcacacatcaatgc	170	
	MVK/8	tgagttcagtgtggacctgc	taattgtgtcctggccttcc	181	
	MVK/9^2^	gaacacctcctccctccac	ttctgagcacagccagattg	295	
	MVK/10	aagtgggaacagatggaacct	ccaatgaggaagcaaagacc	346	
	MVK/11	gtcaagggtgacctgccttc	gcctctccagcagtgtcag	300	
	NLRP3/3a	gatgtgtgtatactttccccctaa	aaacctgtcttggtagagtgtcc	399	
	NLRP3/3c	agcctcatcagaaagaagctg	tgaggtcggactcctcaaac	469	
	NLRP3/3d	tctttggctgcagatggaat	ttgctgagagatcttgcaactta	399	
	NLRP3/3e	tttcctctttggcctggtaa	agaagaagctggcgaggaag	477	
	NLRP3/3c^3^	gcctctctgctcatcac*g*ac	gctcttgccactctccatct	264	
	TNFRSF1A/2	ttgatggtgtctcctctatctga	aagaagcagcaccccagac	240	
	TNFRSF1A/3	gggctccttccttgtgttct	cacatagacaggcacccaca	247	
	TNFRSF1A/4	agaaatgggtcaggtggagat	gccagagaggagttggttgt	280	
qPCR					
	MVK/9^2^	accagccgttccttcttttt	ttctgagcacagccagattg	230	
	NLRP3/2	ggggtctcctctctcatgc	ctagaagcaccaccccagtc	242	
	NLRP3/4	tcgaggctgatttcttttctg	gcactcacacagatcacatgc	248	
	NLRP3/5	tctgatgctttctgcctctgt	cgctggcagaacttccttag	244	
	NLRP3/6	tgactgacattctgccatctct	tctggtaagacacccatgaaga	227	
	NLRP3/7	ggaacagctgggtactgagg	cttgtggccactctgccta	295	
	NLRP3/8	aatcaccccctttttgcag	agagccatcctggattttga	274	
	NLRP3/9a	agtgcaacccaggctttcta	tcacagagctgtggtcttgg	240	
SQF-PCR^4^					
	GFAP/3	gaggaaaggattgatggcca	gaggaggagatccggttctt	249	Control (A to H)
	DMD/28	tgcattttgaattacctgctaca	agtaccaaatagaagacaaatccaaag	361	Control (A to H)
	MVK/2	tattatgatgggcttgaactagg	tgcctcagggtgtcctttta	292	A
	MVK/3	cttcttagcacgtgggtcct	ctctctgtaggctctt	286	A
	MVK/4	gtcgattttctgtgttctgttgtt	agagcatgtgcattctccag	337	B
	MVK/5	ctggaccagatgcttggagt	aagccacgtccctgtcctg	336	A
	MVK/6	gagtggacttgttctttctgagc	agactcttgggcacctacca	328	B
	MVK/7	ttcctgaatggggcaaaat	ctgcctcctatggtacttccc	266	C
	MVK/8	gtatcagggtgggcggcttcc	gagggagacctcgaaaatcc	279	A
	MVK/9	ggctgtgtgaacacctcctc	cacagccagattgcagagcca	314	B
	MVK/10	tctccagccaacaactgtca	tcaagggaattctccaggtg	348	C
	MVK/11	ctgggcttttgccttgaat	aataatccagaaaggggcatc	304	C
	TNFRSF1A/1	accaggccgtgatctctatg	cactcttccctttgtccctg	224	H
	TNFRSF1A/2	aggacttgagccagggaagt	tttccttggggacacacact	198	D
	TNFRSF1A/3	ctggctgttgtccctagcat	cacccacacaccactcaaga	256	E
	TNFRSF1A/4	agaaatgggtcaggtggagat	ttggttgtcagacccacaga	268	D
	TNFRSF1A/5	acaaccaacttcctctctggc	atctgttgcccagctaatgg	277	I
	TNFRSF1A/6	caccagtgccgtctcttctt	atagatggatgggtgggatg	186	I
	TNFRSF1A/7	aacacctgctttgtctgcag	accttctgcccagagtccc	268	H
	TNFRSF1A/8–9	gggaaatcgacacctgaaaa	aagctccccctgaaagagag	233	H
	TNFRSF1A/9–10	cccttcagaagtgggaggac	gatcgatctcgtggtcgct	305	I
	NLRP3/1	ccagagccttcagtttggag	aggagtgtgtcctgagccat	269	G
	NLRP3/2	ccactgtgatatgccaggaa	gcattccaaagagcaggaac	208	F
	NLRP3/3	gcatctcaggtggatgtgtg	caggctcagaatgctcatca	307	G
	NLRP3/4	cctcacttccagtttttgcc	gcactcacacagatcacatgc	189	G
	NLRP3/5	tctgtgtgtgggactgaagc	ctttccccacgacaaacact	228	F
	NLRP3/6	tcagtattgagcaccagcca	tgaccaaagtaacccccatc	213	G
	NLRP3/7	gagtcaaagcagctgcacaa	ccaccatgtgttctcattgc	283	F
	NLRP3/8	gggatggttaaggggacatt	caggcccaacctaatcttga	339	G
	NLRP3/9a	tgcaacccaggctttctatt	tgctgtcattgtcctggtgt	348	F

1 All primers (except HRM-qPCR MVK/9 primer) showed a PCR efficiency range between 80 and 100% (IC+/−0.2%).

2 qPCR and HRM of *MVK* exon 9 could not be performed in the same run due to low PCR efficiency. We used therefore two different primer pairs.

3 p.T348M allele specific primer.

4 Forward primers were 6FAM labeled. Nine distinct PCR sets (Multiplex A to I) were designed, each yielding a pattern of 3 to 6 peaks including the 2 control fragments (DMD and GFAP).

### Assay conditions

qPCR-HRM and qPCR were performed in a single run using a LightCycler 480 instrument (Roche diagnostics, Meylan, France). Reaction mixtures contained 10 ng genomic DNA, 0.25µM of each primer, 3mM MgCl2 and 1× LightCycler 480 High Resolution Melting Master Mix (Roche Diagnostics, Meylan, France) containing Taq polymerase, nucleotides and the dye Resolight in a total volume of 10µl. We used the same cycling conditions for all reactions: an activation step at 95°C for 10 min followed by 50 cycles of 95°C for 15s, a touchdown from 65 to 55°C for 15s (0.5°C/cycle) and 72°C for 20s. Following amplification, products were heated to 95°C for 1 min and cooled to 40°C for 1 min to favor heteroduplex formation. For the melting step, the temperature was raised from 75°C to 97°C with a ramp rate of 0.02°C/s and 25 acquisitions/°C. All reactions were performed at least twice. Each experiment included male or female wild-type DNA (for quantitative calibration and as a qualitative negative control), and positive control DNA with a known point mutation (as a qualitative positive control). All controls are summarized in Table 2.

**Table 2:** Controls used in this work.

	qPCR-HRM						SQF-PCR		
	HRM (Qualitative)			qPCR (Quantitative)			(Semi-Quantitative)		
**Validation Set**									
Negative Controls	N = 48	Wild-type DNAs		N = 10	Asymptomatic individual		N = 10	Asymptomatic individual	
Positive Controls	N = 49	DNAs with known punctual mutations:		N = 3	DNAs with or mimicking a CNV†:		N = 1	DNA with a deletion	
		*MVK* (N = 19)	p.S52N^(1)(2)^,c.371+8C>T^(1)(2)^, p.S135L^(1)^, p.S135S^(1)^, p.D170D^(1)(2)^,c.632−18A>G ^(1)(2)^, c.769-7_769-6dupT^(1)^ *,c.885+24G>A^(1)(2)^, p.G309S^(1)^, p.S329N^(1)^, p.V377I^(1)(2)^, p.R388X^(1)^, p.E296GfsX14^(1)^		*MVK* (N = 1)	Chr12q24.1del		*MVK* (N = 1)	Chr12q24.1del
		*NLRP3* (N = 23)	c.278−45T>C^(1)^, p.Y141Y^(1)^^*^, p.R168Q^(3)^, p.V198M^(1)^, p.T219T^(1)^, p.A242A^(1)(2)^ *, p.P340P^(1)(3)^, p.L344L^(1)(3)^, p.A439P^(1)^, p.H463H^(1)^, p.T587I^(1)^, p.Q703K^(1)(2)^, p.Y859C^(1)^,c.3005+25C>T^(1)^, p.T348M^(1)^, p.L411L^(3)^, p.S434S^(1)(2)^, p.T436P^(3)^		*NLRP3* (N = 1)	p.T348M artificial deletion (allele specific primer)			
		*TNFRSF1A* (N = 7)	p.C30R^(1)^, p.C30F^(1)^, p.C73W^(1)^, p.H69fs^(1)^, p.E54E^(1)^, p.P46L^(1)^, p.R92Q^(1)^		*ITGB5* (N = 1)	Chr3q21.2dup			
**Study group**									
Negative Controls	N = 1	Wild-type DNA		N = 1	Asymptomatic individual		N = 1	Asymptomatic individual	
Positive Controls	N = 1	DNA with a known variation per exon tested where available		N = 1	*DMD* (Internal control)		N = 1	*DMD* (Internal control)	

^(1)^ heterozogote, ^(2)^ homozygote, ^(3)^ compound heterozygote.

*These polymorphisms were not detected by HRM.

† Copy number variation.

### Qualitative analysis for punctual mutation screening

Upon completion of the run of the qPCR-HRM (approximately 2 hours), HRM curve analysis was performed using the LightCycler 480 Software version 1.5 supplied with the device. The melting curves were normalized and temperature shifted to allow the direct comparison of samples. Difference plots were generated by selecting a negative control, converting the melting profile to a horizontal line and normalizing the melting profiles of the other samples against the test sample. Significant differences in fluorescence from the horizontal baseline were indicative of mutations. Differences were judged as significant if the replicates fell outside the range of variation seen in the wild type samples.

### Quantitative analysis for sequence rearrangement screening

Standard curves were generated for all exons investigated (Figure 1) and for two reference genes (exon 16 of the X-linked gene *DMD*, and exon 2 of *B2M*) to derive sample and calibrator (negative control) relative DNA amounts. The ratio (R) representing the relative copy number of the target exon in the patient was automatically calculated by the LightCycler 480 data analysis software (LC480; Roche) according to the formula:
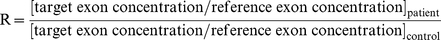
The gene *DMD* was analyzed in each experiment as a control of correct gene dosage: the ratio *DMD*/*B2M* should be equal to two for female samples, and one for male samples.

### SQF-PCR

#### Primer design

The method of SQF-PCR is based on the comparison of fluorescent profiles of multiplex PCR fragments obtained from different samples (patients and control)^4^. Primers labeled with 6FAM fluorochrome (Table 1) for exons 2–10 of *MVK*, exons 1–10 of *TNFRSFA1*, exons 1–9 of *NLRP3*, and 2 internal control genes (exon 28 of *DMD* and exon 3 of *GFAP*) were designed using the PrimerBlast software (http://www.ncbi.nlm.nih.gov/tools/primer-blast/). We chose allele pairs with the same annealing temperature to perform multiplex PCR resulting in fragments of sizes differing by at least 20bp (186–361 bp). We checked that all exons were adequately co-amplified.

### Assay conditions

The PCR reactions were performed using the QIAGEN Multiplex kit (Qiagen, Courtaboeuf, France) in a total volume of 20 ul containing 100 ng of genomic DNA and 5 to 20 pMol of each primer. The amplification was stopped at the exponential phase (22 cycles). The PCR cycling conditions used were those suggested in the manufacturer's instructions. The PCR products were then separated on an ABI3130xL DNA Sequencer (Applied Biosystems, Foster city, CA, USA).

### Semi-quantitative analysis

Electrophoregrams were analyzed using the GeneMapper software v4.0 (Applied Biosystems, Foster City, CA, USA). In each experiment, the genes *GFAP* and *DMD* were analyzed to determine the relative gene dosage for *MVK*, *NLRP3* and *TNFRSFA1*. To calculate the relative copy number (R) of each target region, each peak value including height and area from the patient samples was compared with those from the normal control after normalization with the reference gene *GFAP*. The data (area and height of peak) were exported to Microsoft Excel and inserted into the equation:
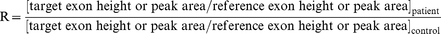
The *DMD* gene was used as a control of correct gene dosage: the ratio (R) *DMD*/*B2M* should be equal to two for female samples and one for male samples.

## Results

We first implemented qPCR-HRM as a valid method for screening hereditary recurrent fever genes in our laboratory. Then in our patient cohort, we conducted a simultaneous search for punctual mutations and gene rearrangements using qPCR-HRM in the prospective group and qPCR alone in the retrospective group (as these patients had already been assessed for qualitative alterations), then SQF-PCR in both groups as a confirmatory quantitative approach (Figure 1).

### Validation of qPCR-HRM

To implement a strategy that could be used on a routine basis, we set the method up to focus on mutation hot spots (*MVK*, exons 2–11; *NLRP3*, exon 3; *TNFRSF1A*, exons 2–4). The qPCR-HRM method could be performed for all regions except *MVK* exon 9 for which the Ct could not be obtained below 30 cycles. For this exon, we therefore conducted separate HRM and qPCR. The validation process included a number of DNA controls to assess the successful detection of a variety of gene alterations.

To validate the mutation analysis (HRM), we used 49 DNA samples carrying known mutations and 48 wild-type controls (Table 2). The sensitivity of the technique in our conditions (93.8%) was comparable to other mutation screening methods [9]. Indeed, all pathological mutations (homozygous or heterozygous) were detected, and only 3 polymorphisms (c.769-7_769-6dupT in *MVK* and p.A242A, p.Y141Y in *NLRP3*) went undetected (not shown).

**Figure 2:**
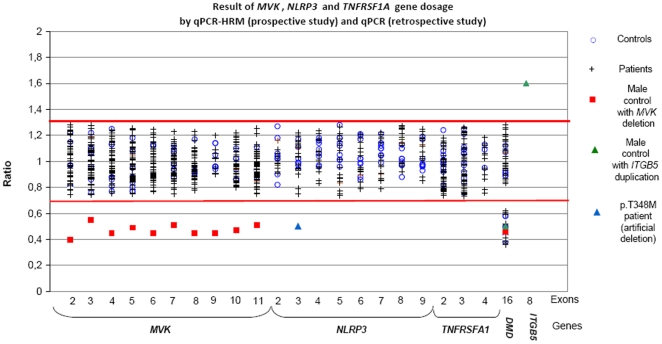
Results of *MVK*, *NLRP3* and *TNFRSF1A* gene dosage analysis by qPCR-HRM (prospective study) and qPCR (retrospective study). We screened by qPCR-HRM (i) exons 2–8 and 10–11 of the *MVK* gene in 10 controls, 1 DNA sample with a 12q23.2 deletion, and 49 patients, (ii) the exon 3 of the *NLRP3* gene in 9 controls, 10 patients, and the artificial positive p.T348M patient and (iii) exons 2–4 of the *TNFRSF1A* gene in 13 controls and 54 patients. We screened by qPCR (i) exon 9 of the *MVK* gene in 10 controls, 1 DNA sample with a 12q23.2q24.11 deletion, and 12 patients and (ii) exons 2–9 of the *NLRP3* gene in 9 controls and 38 patients.
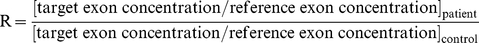
The red bars indicate the range limits of the control ratios, between 0.75 and 1.28.

To validate the gene dosage (qPCR), we used DNA samples from 3 individuals (Table 2): 1) One patient with no recurrent fever in whom was found a large deletion (2,13Mb, Chr12:107813096..109948089, version: NCBI36/hg18) including the *MVK* gene in an unrelated study (micro-array screening for mental retardation); 2) One patient with a p.T348M (c.1043C>T) mutation in *NLRP3* in whom we performed allele specific PCR using a wild-type primer to mimic a deletion; 3) One patient with 3 copies of the gene *ITGB5* (154.9kb, Chr3:125946672..126101558, version NCBI36/hg18), since we had no available sample with a duplication in our genes of interest. As normal controls, 10 DNA samples from asymptomatic individuals were screened. Expected copy numbers were obtained (Figure 2).

#### Prospective study: qPCR-HRM has little relevance for routine genetic diagnosis of hereditary recurrent fevers

**Figure 3:**
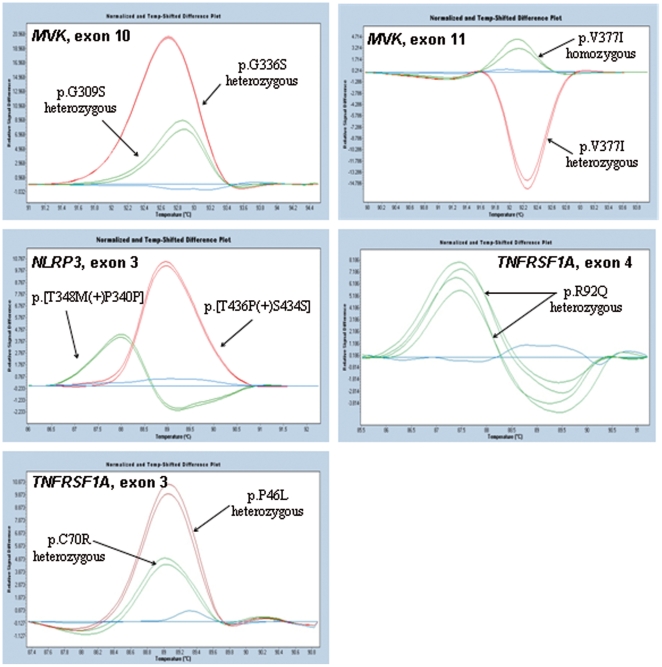
Pathological genotypes detected by qPCR-HRM in the prospective study. Qualitative changes were assessed using the gene scanning module of the LightCycler 480 Software version 1.5. Wild type sequences were used to define baselines. Difference plots revealed disease associated variants.

**Figure 4:**
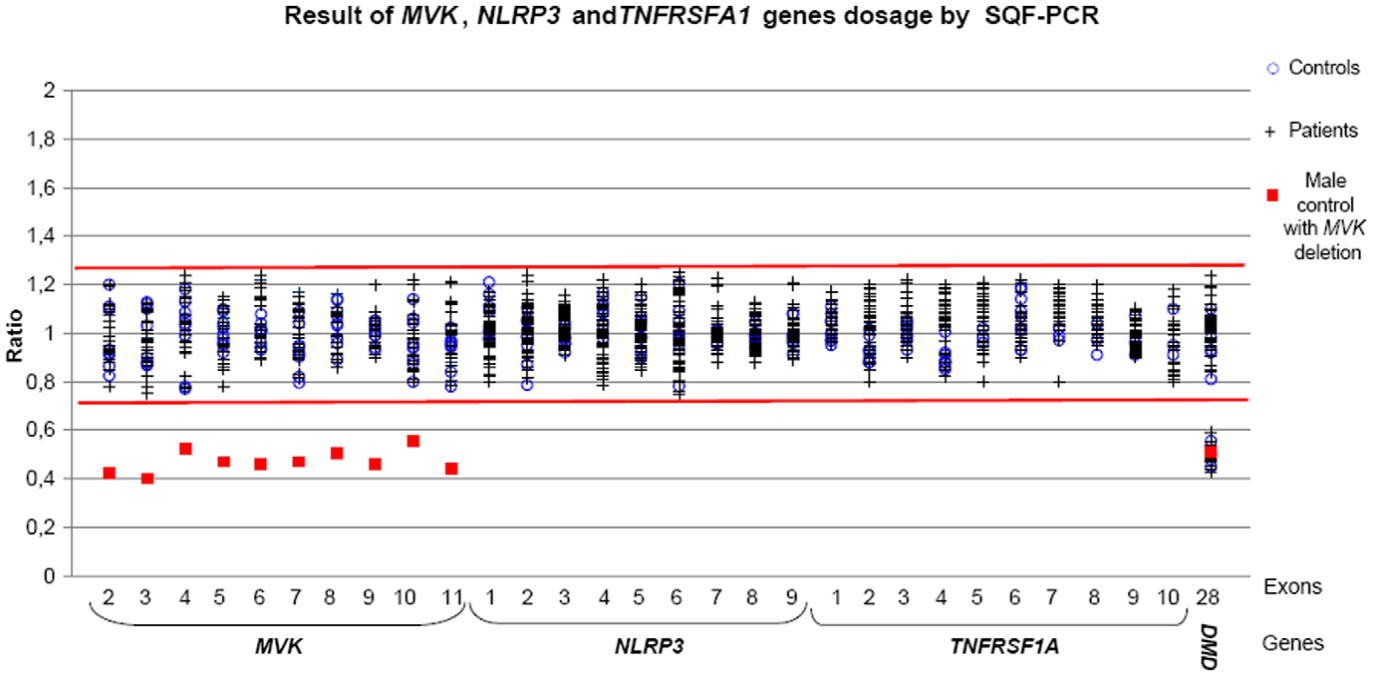
Results of *MVK*, *NLRP3* and *TNFRSF1A* gene dosage analysis by SQF-PCR. We screened by SQF-PCR (i) all coding exons of the *MVK* gene in 9 controls, 1 DNA sample with a 12q23.2 deletion, and 28 patients (ii) all coding exons of the *NLRP3* gene in 9 controls and 86 patients and (iii) all coding exons of the *TNFRSF1A* gene in 9 controls and 54 patients.
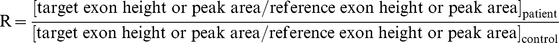
The red bars indicate the range limits of the control ratios, between 0.75 and 1.25.

To assess the usefulness of this approach for routine genetic diagnosis, we applied it to our prospective study group consisting of 113 unselected patients referred for genetic diagnosis. All amplicons were sequenced in parallel. We confirmed diagnosis in 6 patients, all of whom bore already known mutations: three associated with MKD, p.[V377I]+[V377I], p.[V377I]+[G226S], and p.[T237S]+[T237S]; one with CAPS, p.T348M; and two with TRAPS, p.C70R and p.R92Q. Representative HRM difference plots are shown in Figure 3. The qualitative step (melting curves) detected all the mutations except the T237S homozygous genotype which was only detected after equimolar mixing with a wild type sample using the HRM method alone (not shown). The quantitative step detected no gene dosage abnormalities (Figure 2). A second method (SQF-PCR) was performed on a subset of these patients and confirmed the absence of copy number variants in this cohort (Figure 4).

#### Retrospective study: sequence rearrangements are likely absent in hereditary recurrent fever genes

To confirm this absence of copy number alterations in our genes of interest and enhance the probability of finding new mutations, we extended our series to a panel of selected patients with recurrent fever (N = 88, Figure 1) with either one *MVK* punctual mutation for MKD, or urticaria and/or neurosensorial symptoms for CAPS. Indeed, FMF had previously been addressed^3^ and TRAPS does not show a clear cut phenotype. In this retrospective series, punctual mutations in all coding exons and intron boundaries had already been investigated using classical one-step routine analysis (HRM and/or sequencing). No genetic rearrangements were identified (Figure 2). We also performed a second SQF-PCR method in this retrospective panel which confirmed the absence of intragenic duplications or deletions (Figure 4).

## Discussion

The existence of a significant number of patients with convincing symptoms though not carrying point mutations suggests that other genes and/or mutational mechanisms, such as gene dosage alteration, could be involved in the etiology of hereditary recurrent fever syndromes. The aim of this study was therefore to explore the possible existence of copy number variants in hereditary recurrent fever genes and estimate the usefulness of qPCR-HRM in routine diagnosis. Duplications and deletions of the genes involved in MKD, a recessive enzymopathy [10], and TRAPS, thought to be a protein misfolding disorder could theoretically lead to these two diseases [11]. CAPS is due to gain-of-function mutations in NLRP3, a major protein of the inflammasome platform that activates pro-caspase-1 into its active form caspase 1, which in turn promotes the processing of the potent pro-inflammatory cytokine interleukin-1β [12]. Classical gain-of-function rearrangements (e.g. duplications) have never been recorded in CAPS. However, a nonsense mutation (p.Arg554X) was identified in *NLRP3* putatively resulting in a truncated protein lacking all leucine-rich repeats [13]. A partially deleted protein could therefore result in a gain-of-function.

To address the issue of possible pathological copy number variations in hereditary recurrent fevers, we took advantage of the fact that the HRM mutation screening technique is already routinely used in our laboratories, and implemented a recently described strategy that combines point mutation and gene rearrangement detection in a single assay (qPCR-HRM). This approach proved useful for the genetic analysis of *MLH1* in Lynch syndrome [7]. The main advantage of qPCR-HRM is the gain in terms of cost and time. Another major benefit is the validation of amplification of the two alleles through the quantitative measure, which avoids the misinterpretation of primer mismatch of one allele as a deletion, and validates homozygous genotypes. We found the sensitivity of qPCR-HRM for qualitative analysis as compared to sequencing (93.8%) to be similar to that of classical qualitative methods such as DGGE or dHPLC [9]. The sensitivity of qPCR-HRM and SQF-PCR for quantitative analysis was 100% for the positive controls. The known limit of qPCR-HRM is the difficulty in designing primers efficient for both qualitative detection and quantitative dosage. Among the 21 amplicons we designed, only exon 9 of *MVK* could not be assessed by qPCR-HRM.

Having validated this new method being in our laboratories, we then used it to assess our patients in a prospective and retrospective study. We detected no gene dosage alteration in a total of 159 patients suspected of MKD, CAPS or TRAPS. A previous study in patients with FMF using MLPA also found no genetic rearrangements. A recent review demonstrated that copy number variants are more frequent in large genes [14]. The hereditary recurrent genes are in contrast rather small (*MVK*: 23434bp, *NLRP3*: 31054bp, *TNFRSF1A*: 13231bp). To investigate whether the chromosomal regions containing these genes could contain fragile sites, we consulted the Database of Genomics Variants (http://projects.tcag.ca/variation) and the Copy Number Variation project at the Children's Hospital of Philadelphia (htpp://cnv.chop.edu). We retrieved no copy variant within the regions of the *MVK* and *TNFRSF1A* genes. However, 3 variants have been reported in apparently healthy individuals in the 1q44 region of *NLRP3*, the largest of the 3 genes: 1) an insertion of 147bp in intron 3 of the *NLRP3* gene (position 245657612 on chromosome 1q, version NCBI36/hg18) in one Caucasian individual [15]; 2) a deletion spanning intron 4 to the 5′extremity of exon 5 (chr1:245663794..245664071) in one Asian individual [16]; and 3) complex rearrangements upstream of the *NLRP3* gene in 50 French individuals [17]. This may indicate the localization of *NLRP3* in or close to a minor fragile site, although we identified no pathological rearrangements in our series.

In conclusion, our previous and present results support that deleterious intragenic rearrangements in *MEFV*, *MVK*, *NLRP3* and *TNFRSF1A* are rare or absent from the mutational spectrum of hereditary recurrent fevers, suggesting that other mutations in seemingly affected patients should be searched for either in other gene regions (e.g. promoter and introns) or in other candidate genes. The use of the combined qPCR-HRM as a first line approach for genetic diagnosis is not relevant for these diseases since only qualitative alterations were identified. However, rare pathological gene dosage alterations could still be found in future series and the method we have developed could be used punctually as a second approach since it proved effective and quick.
